# From the Cyclone Idai disaster to the COVID-19 pandemic: An account of inadvertent social capital enhancement in Eastern Chimanimani, Zimbabwe

**DOI:** 10.4102/jamba.v13i1.1068

**Published:** 2021-11-29

**Authors:** Wisemen Chingombe, Happwell Musarandega

**Affiliations:** 1 School of Biology and Environmental Sciences, Faculty of Agriculture and Natural Sciences, University of Mpumalanga, Mbombela, South Africa; 2 Department of Geography and Environmental Science, Faculty of Agriculture and Natural Sciences, University of Fort Hare, Alice, South Africa

**Keywords:** COVID-19, Cyclone Idai, resilience building, social capital, socio-economic, meteorological disaster, disaster response, Chimanimani

## Abstract

Zimbabwe suffered a devastating meteorological disaster when Cyclone Idai affected the southeast part of the country in March 2019. Barely a year after the cyclonic event, the coronavirus disease 2019 (COVID-19) pandemic emerged, leading to the declaration of a nationwide lockdown that paralysed socio-economic systems. This article examines how social capital was autonomously cultivated and eventually utilised by the Cyclone Idai disaster survivors in Eastern Chimanimani to face the fresh socio-economic challenges of the COVID-19 pandemic. In this article, a qualitative method embedded in a case study design was used. Data was collected using 30 purposively selected key respondents who interacted with victim communities from March to July 2020. A thematic content analysis approach was applied to obtain opinion patterns and subsequent inferences. The study results revealed a lack of immediate external disaster intervention during the Cyclone Idai disaster in Chimanimani. Accordingly, a strong sense of collective action developed between victim communities, thus enabling them to perform hasty operations meant to salvage lives and property. The enhanced social capital helped the Cyclone Idai victims to face the new COVID-19 lockdown challenges. This article recommends pro-active and well-coordinated government and private sector disaster response strategies supporting local area initiatives to minimise loss of lives and property during disaster situations.

## Introduction

The Southern African region is home to the world’s most climate-vulnerable countries (The Intergovernmental Panel on Climate Change [IPCC] 2014; Marigi 2017; Shackleton, Ziervogel & Sallu 2015). Zimbabwe is one such country that has often experienced severe climate-related disasters over recent years. The disasters range from persistent drought to excessive rainfall coupled with severe flooding, thunderstorms, and devastating winds (IPCC 2014; Manatsa et al. 2013; Shackleton et al. 2015). Such natural disasters are tortuously connected to the heightening of human vulnerability in many community settings (Mavhura, Manatsa & Matiashe 2017). Climate change is related, amongst other negative trajectories, to the emergence and proliferation of complicated infectious diseases (IPCC 2014; Ncube & Tawodzerwa 2019). The subsequent disease complications call for well-coordinated efforts by governments, the private sector, non-governmental organisations (NGOs), and communities to combat them.

In March 2019, Zimbabwe experienced Cyclone Idai that disrupted the social functioning of human communities, particularly in the eastern part of the Chimanimani district, where the cyclone had devastating effects (World Bank 2019). The cyclone killed over 340 people, displaced 51 000, whilst many other victims went missing (OCHA 2019). The disaster damaged approximately 634 km stretch of roads, 140 schools and 1481 homes (Chatiza 2019). It destroyed common survival forms, including food security, education, health, and other facets of human existence (OCHA 2019). The disaster occurred when communication networks had been cut off because of the cyclone’s earlier effects. Subsequently, Eastern Chimanimani was cut off from the rest of the world. External intervention efforts became impossible.

Just a year after Zimbabwe had the deadly Cyclone Idai, another fatal health pandemic, the coronavirus disease 2019 (COVID-19), followed. The coronavirus disease 2019 is a respiratory infection caused by a viral attack. It was first reported from Wuhan City in China (Food and Agricultural Organization [FAO] 2020) at the end of 2019, and quickly spread to many parts of the world. The COVID-19 virus is transmitted through droplets, aerosol, and oral-faecal routes through social contact networks (Zhao 2020). In recognition of the disease’s related fatalities, the Government of Zimbabwe gazetted and unveiled Statutory Instrument 83 of 2020 on Public Health (COVID-19 Prevention, Containment, and Treatment) (National Lockdown) Order, 2020. Subsequently, a nationwide lockdown was declared on 30 March 2020 (Mackworth-Young et al. 2020).

Compared to the Cyclone Idai disaster, the COVID-19 pandemic disturbed socio-economic systems across the nation. The Statutory Instrument compelled people to stay in their homes throughout the lockdown period. The imposition of a strict nationwide lockdown reminded the people of Chimanimani of the Cyclone Idai experiences. Livelihoods were already disturbed. Added to the high statistics of fatalities, propagated news of adverse COVID-19-related deaths in other parts of the world, residents of Chimanimani were quickly reminded of the Cyclone Idai fatalities, as the socio-economic challenges accompanying COVID-19 were almost the same as those of the cyclone.

This article uses the social capital theory as the guiding framework to analyse the enhancement of social capital by the people of eastern Chimanimani as they moved from the Cyclone Idai trauma to the COVID-19 lockdown. Social capital is a package of obligations and connections within members of a group (Andriani 2013; Van Breda 2018). According to Norris et al. (2008:127). It implies the received and expected social support, reflected in citizen participation and a general sense of attachment to one’s community. Also, we strongly advocate in this article that resilience to environmental adversity by communities is best accomplished when members of the community have good social relations amongst themselves. Although it is hardly quantifiable (Bhandari & Yosunobu 2009; Andriani 2013; Van Breda 2018), social capital forms the mainstay of several other forms of capital in society, such as financial and physical capital. When social relations are strengthened, it is easier for societies to develop functional economic structures as their social relations help pull them together to share valuable business ideas.

In addition, we specifically chose the social capital philosophy as an underpinning analytical tool because our goal was to explain how cooperation and community trust between people can be harnessed for socio-economic development. Delic, Šaric and Osmanovic (2017) advocated for such an analytical approach when dealing with groups of people undertaking collective action and belonging to common solidarity networks. In addition, social capital enhancement plays a critical role within poverty-ridden communities, particularly in the backdrop of limited outside assistance (Delic et al. 2017; Marango 2011; Shackleton et al. 2015).

By drawing insights from Tsholotsho Cyclone Dineo’s experiences, Mhlanga, Mzingili and Mpambela (2019) asserted that social work interventions in Zimbabwe are generally limited before and during disaster events. Consequently, locally based initiatives become critical in saving lives during disasters in the absence of immediate external intervention. Accordingly, this article proposes exploring the package of social capital enhancement that the people of eastern Chimanimani experienced from Cyclone Idai, which helped them hold up the consecutive challenges instigated by COVID-19 lockdown restrictions.

## Methodology

The study was conducted in the far eastern part of the Chimanimani District in Zimbabwe. A case study design focusing on community settings was used. The selected communities are: Ngangu, Rarthmore, Charleswood, Manase, Chisengu, Chikukwa, Charter, Ndima, Thorndon, Cambridge, Machongwe, Nyabamba, Dzingire, Rosecommon, and Ndakopa. The selection criterion was based on the fact that these district were severely affected by Cyclone Idai in March 2019.

A total of 30 liaison persons with close connections to the 15 community settings were purposefully selected and used as rapporteurs and key informants. These comprised 15 community healthcare workers and 15 members who actively participated in Cyclone Idai voluntary rescue operations. To pick the 15 voluntary participants, we randomly visited some households. We asked for the names of ‘main actors’, that is, those who outstandingly participated in the voluntary rescue and other operations during the Cyclone Idai. The names of such ‘heroes’ could easily come up such that in each case, we narrowed down to the most frequently identified individuals. When approached and asked to collect sentiments from victim communities, the 15 members agreed and willingly participated in the study. The other 15 liaison persons were drawn from the pool of community healthcare workers operating in the area. We considered them because Cyclone Idai and COVID-19 strongly impacted people’s health and lives. Our selection was also based on the fact that healthcare workers know the area quite well. The local people also trust them as they frequently shared highly confidential health and socio-economic welfare matters.

To select the liaison persons, no strict ratio was used. However, we considered the skewed nature of the population in terms of the male and female distribution. The district has more females than males (ZIMSTATS 2016). Accordingly, we used six males and nine females for each group, that is, the volunteer rescuers and community healthcare workers, to make a total of 30 liaison persons. These key informants were provided with a set of open-ended interview questions and ample time to interact with victims of Cyclone Idai between March and July 2020, the period during which the COVID-19 lockdown was already in full force. The questions did not remain rigidly positioned, but acted as guidelines (Cutter, Burton & Emrich, 2011; Macchi 2011) with a view to source as much data concerning both Cyclone Idai and COVID-19 as possible. Also, the liaison persons were not restricted to specific numbers of participants because we intended to collect as much qualitative data as possible (Saunders, Lewis & Thornhill 2015). Data collected from participants were qualitatively analysed using the thematic content analysis technique. Thus, the sentiments from various participants were collected, scrutinised, and then clustered according to their likeness to draw valuable conclusions.

Necessary ethical considerations were put in place. Permission to enter villages and compound settlements was obtained from related Ward Councillors and traditional leaders. Given the COVID-19 pandemic, social distancing and sanitation protocols as defined in the regulations set out by the Government of Zimbabwe following World Health Organization (WHO) regulations were strictly adhered to. As a result of the sensitivity of the views regarding Cyclone Idai deaths, property loss and related feelings and sentiments, we ensured that perceptions drawn from participants were kept highly confidential. Under eighteen adolescents were not considered as interviewees because the study was ethically designed not to consider those regarded as minors.

## Results

**FIGURE 1:**
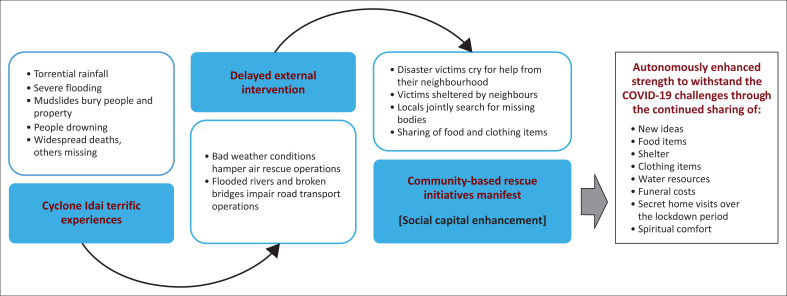
Conceptual analysis of community capital enhancement in Chimanimani during and after the Cyclone Idai disaster.

We aggregated sentiments from the participants regarding social capital enhancement in eastern Chimanimani from Cyclone Idai to the COVID-19 pandemic. Figure 1 shows an analytical model of social capital enhancement using information gathered from the key informants regarding the Cyclone Idai natural disaster and the COVID-19 pandemic.

The socio-economic isolations effects experienced by the victims of Cyclone Idai were more or less similar to those that accompanied the COVID-19 lockdown. When floods destroyed roads and bridges in eastern Chimanimani, people were cut-off from the rest of the world. The general disconnection from the outside world typified the lockdown scenario that COVID-19 brought. Concurring sentiments from the participants indicated that before Cyclone Idai, people were not as closely connected as they became after the tragedy. However, the floods and landslides disturbed the subsistence farming of food crops like maize, yams, beans, and vegetables. There was an overall decrease in commercial crop yields like bananas, hence significantly impacting people’s livelihoods. Those thriving in the transport industry were concomitantly affected when agricultural produce plummeted for extended periods. Most banana fields were destroyed in Ndima, Ngorima, Machongwe, and Chikukwa leading to a slow recovery trajectory.

Consequently, the Cyclone Idai disaster culminated in severe dependency on food handouts from well-wishers for quite some time. In a way, community social relations unconventionally became enhanced. Disaster victims could not immediately pick up food and other material handouts from the government and humanitarian organisations because of the physical inaccessibility of the region. During interviews, survivors repeatedly mentioned statements like ‘we sought help from our neighbours’, ‘we dug the rubble to look for dead bodies’, and ‘had it not been our fellow members’. We, therefore, used such insights to interrogate participants on the impact of the disaster on their social relations during and post the Cyclone Idai disaster period. We realised that relations got enhanced as people came together to deal with the catastrophe. An older man from Chinyaeni village, whose family went through the disaster, narrated:

We could not stop shouting for assistance from our neighbourhood irrespective of our previous relations. After floods destroyed our main house and ban, we sought help from our next-door neighbour, who provided food and water. NGOs and other aid agencies took a long to come, so many victims of the disaster survived in that way. During this Coronavirus lockdown, people exchange essential commodities because it is difficult to reach out to major centres like Chipinge town and the City of Mutare.

After Cyclone Idai, floods and landslides occurred; family setups were violated, leaving orphans and widowed spouses. Consequently, a new wave of family integration manifested. Desperate members from disaster-broken family structures were incorporated into new family structures in their neighbourhood. Thus, extended separated lineage members unexpectedly reunified and revived their social relations. The development manifested out of the desperate efforts to integrate stranded disaster victims from traumatised households. In some extreme instances, stranded strangers reportedly joined unknown households and became part of their family for prolonged periods. One elderly survivor stated that every human being either familiar or unfamiliar became a close acquaintance during and soon after the Cyclone Idai disaster. He added that food and other belongings in the house were for the community because the situation at hand was terrible.

The culture of sharing ideas and resources during problematic situations reportedly stretched through to the COVID-19 period in 2020. When the government of Zimbabwe declared a nationwide lockdown, some families did not have adequate food and other material stocks. It was reported that many families benefited from the relations that they had established during and after Cyclone Idai. Already, a network of lending and borrowing had been cultivated in eastern Chimanimani localities. Some Cyclone Idai victims in temporal structures reportedly moved out to join their relatives in resettlement areas outside Ngangu and Kopa as they feared the spread of COVID-19.

The gruesome experiences associated with Cyclone Idai in 2019 equipped the local people with the emotional strength to face the new challenges brought by the COVID-19 pandemic. Major employers in the region, such as Charter Factory, Allied Timbers, and Chisengu Estate, scaled down their operations, forcing most employees to succumb to subsequent remuneration slashes and severe economic stress. For many such victims of joblessness, the remaining viable option became that of re-thinking new ways of survival under COVID-19 lockdown. Cyclone Idai that had forced surviving victims to initiate subsistence projects like gardening, piggery, chicken rearing, and trading various available commodities quickly adapted to the new COVID-19 survival challenge. One surviving victim of the Cyclone Idai flood in Chinyayeni village commented that in her village, people moved from Cyclone Idai lockdown to COVID-19 lockdown, meaning there was nothing new for them. She had this to say:

‘Cyclone Idai tormented us to extreme levels. Before we had even recovered, COVID-19 followed. We never had a chance to recover but simply moved from one challenge to the other. Now, these COVID-19 lockdown restrictions are protracted and repressive, and we never know when these will end. It appears like Cyclone Idai came to prepare us for a much more extended period of suffering from an unidentified end.’ (Cyclone Idai survivor, woman, Chinyayeni village)

The woman implied that the Cyclone Idai hardships ruthlessly and unconsciously prepared the villagers to face the COVID-19 lockdown. In addition, the woman said that those without resources to start their subsistence engagements relied on their neighbours to share preliminary resources like pieces of land and seed.

Whilst many communities were subjected to COVID-19 lockdown restrictions, some sections capitalised on the social networks developed during and after Cyclone Idai, and therefore met and worshipped in small cohorts averaging three families. During and long after Cyclone Idai, people skipped attending church and other public reverence services as they waited to recuperate from the intense psychological trauma. Similarly, public gatherings were officially cast out as part of the COVID-19 lockdown restrictions. People’s religious beliefs, including their worship patterns, became discreet but more strongly conjoined after Cyclone Idai than they were before. A broad range of sentiments from interviewed participants surprisingly revealed that people developed close social and spiritual ties that empowered them to grapple with the trauma inculcated by Cyclone Idai. An elderly couple in Ngangu reiterated that the solid social collectivisation that grew during Cyclone Idai calamity enabled cyclone victims to grapple with COVID-19 lockdown challenges.

During the Cyclone Idai experiences, victims reportedly set aside their political differences as they urgently united to save lives and property. The reason was that during a disaster situation, human survival receives greater priority than political affiliation. The solid social coordination enabled the surviving members to provide food, psychological support, clothes, and other utilities to those who lost their relatives and property. The deteriorating living standards in Chimanimani generally increased citizen visibility in state politics as several disaster victims increasingly felt that the government agents failed the mandate to protect their livelihoods. Thus, the unfavourable socio-economic and environmental trajectory brought by Cyclone Idai reportedly exposed the political leaders in the area as majority of the people, particularly in Ngangu and Kopa, openly criticised the cases in which few political leaders withheld some humanitarian aid such as food and clothes. With COVID-19, many people realised the significance of collective action as they now value and depend on their neighbourhood irrespective of their conflicting political affiliations.

## Outstanding challenges

However, it should be kept in mind that Cyclone Idai unequivocally intensified the political situation as some unscrupulous political figures compromised the distribution of relief goods and services. The majority (73%) of the 15 interviewed community healthcare workers revealed that some funds and goods were reportedly looted and misdirected on political grounds by some misguided individuals. Moving on to the COVID-19 pandemic, some donated personal protective equipment (PPE) were misappropriated by few imprudent community leaders connected to political heavyweights working with certain humanitarian agencies.

To date, some survivors of the Cyclone Idai disaster who were relocated and temporarily accommodated in crowded makeshift homes are still not provided with permanent, decent homes. Such people are not socially connected and protected from the ongoing socio-economic challenges of the COVID-19 pandemic. The unfortunate development remains destructive to the social fabric before the Cyclone Idai disaster. Most victims still live in tents and makeshift settlements where children’s upbringing is compromised and societal values are fast eroding, mainly in Ngangu residential area. The physical distancing measures meant to reduce the spreading of COVID-19 have not been effective as Cyclone Idai victims continue to leave under crowded conditions rendering these victims vulnerable to yet another risk because of COVID-19.

## Discussion

The Chimanimani study revealed that social networks embedded in communities are inexorably activated in difficult disaster situations such as Cyclone Idai and the COVID-19 pandemic. In the wake of adversity, humans are bound to fight to bounce back because they are naturally endowed with the phenomenal potential to withstand adversity and acclimatise when compelled to do so. Still, they require key material and social resources to achieve that (Southwick et al. 2014). When people are well-connected in their neighbourhoods, it is much easier to pass on crucial information to alert potential victims of impending disasters (Delic et al. 2017). Ncube and Tawodzerwa (2019) were of the same view and asserted that households generally comprise unique and unreceptive social networks transferred across generations and cultures. Mashizha (2019) also noted the significance of harnessing social capital to enhance community resilience to hostile weather conditions such as drought and other adverse events.

The improved social relations enabled some smallholder farmer communities in Chimanimani to quickly recuperate following the Cyclone Idai disaster. They did not sit back after the cyclone destroyed their livelihood sources, hence they are able to face off the COVID-19 lockdown challenges. Although climate-related disasters have intensified because of climate change (IPCC 2014; Mavhura et al. 2017; Mhlanga et al. 2019), the events have occurred since time immemorial. In response, people have always fought back through social capital enhancement to recover their means of livelihood sustenance (Mavhura 2017; Ncube & Tawodzera 2019). After all, civil society can utilise its own formal and informal means of social organisation and collective action separate from government intervention initiatives (Carlson et al. 2012; Mashizha 2019).

The reported delays in rescue and other humanitarian interventions by the government and non-governmental agencies were confirmed in many studies. Chitongo et al. (2019) corroborated this when they alluded that bad weather conditions crippled military evacuation and rescue operations. Chatiza (2019) reiterated that the immediate disaster response came from the Chimanimani victim community as the area was not initially accessible to external intervention. In the backdrop of disasters, social capital plays a significant role because disaster victims easily share the little material remnants. Likewise, the resilient members of the community find it easier to accommodate and comfort those severely affected by the disaster. After all, social capital is a critical tool that communities utilise to build their resilience to emerging challenges (Andriani 2013; Bhandari & Yasinobu 2009; Southwick et al. 2012). Thus, the local people collaborated to form disaster relief networks that helped disaster victims in their neighbourhoods.

Strengthened collective action became inevitable in Chimanimani during and after the Cyclone Idai disaster. In supporting this development, Southwick et al. (2014) asserted that effective disaster intervention with the capability to enhance increased resilience is feasible where parties involved realise the fact that individuals are rooted in families and communities, which in turn are embedded in societies and cultures. This is the norm in several other disaster scenarios. Mashizha (2019) highlighted the case of smallholder farmer communities in Zvimba District, Zimbabwe, who collectively coordinated to fight the adverse effects of drought through the Zunde raMambo (chief’s granary scheme) scheme in which they pooled their labour to produce supplementary food reserves. Victims of the disaster stripped of their property often seek primary assistance from surviving members in the immediate neighbourhood.

The study findings on social capital enhancement are also corroborated by Chazovachii et al. (2019), who recorded the resilience-building initiatives of the poor people in Chimanimani by forming a network of community-based Rotating Savings and Credit Associated Schemes (ROSCAS) following the Cyclone Idai disaster in Chimanimani. Accordingly, Ncube and Tawodzera (2019) alluded that social capital governs human perceptions, knowledge, and community capacities.

The Chimanimani people faced several challenges in their quest to recover from the aftermath of Cyclone Idai. Such trauma has been the case in many other community settings that are vulnerable to disaster prevalence. After all, the resilience trajectory is often preceded by a transitory period of disequilibrium before the resumption of normality (Southwick et al. 2014). Besides, it is argued that social capital is a public good (Andriani 2013; Bhandari & Yasunobu 2009) that relies heavily on the preparedness of community members to desist from free-riding tendencies, a common challenge, particularly in the developing world, where the economic situation makes it hard for victims of natural disasters to recover from disaster shocks and subsequent stressfully. Southwick et al. (2014) also acknowledged that extreme weather conditions have significantly influenced some of the extended stress people have within communities.

## Conclusion and recommendations

Cyclone Idai wreaked havoc in eastern parts of Chimanimani in Zimbabwe. Delayed external intervention by government and non-governmental agencies forced disaster victims to collectively save lives and recover desecrated livelihoods. Before the people fully recuperated from Cyclone Idai trauma, a strict lockdown in response to the COVID-19 pandemic ensued. The social distancing measures that came with the COVID-19 lockdown forcedly isolated communities and perpetuated the halted socio-economic interdependence initially created by Cyclone Idai. Consequently, many people in the affected communities of eastern Chimanimani continue to ride on collective action that developed during and after Cyclone Idai adversity.

Accordingly, this article makes the following recommendations:

- The double tragedy of post-Cyclone Idai and the current COVID-19 lockdown challenge means Chimanimani communities need the state’s and stakeholders’ undivided attention, lest the levels of poverty and vulnerability continue to grow.
- There is a dire need to consider massive funding of the affected community to resuscitate projects to stimulate people’s livelihoods.
- To reduce the impact of catastrophes such as COVID-19, some disease awareness programmes should be implemented to appropriately prepare the local people’s mindsets.
- The government, the private sector, and development agencies should put together relief programmes and counselling units to alleviate suffering from the COVID-19 pandemic.
